# Comparative Tumor Microenvironment Analysis for HCC and PDAC Using KMplotter

**DOI:** 10.3390/ijms262411920

**Published:** 2025-12-10

**Authors:** Wen-Han Chang, Drashya Shah, Scott Myers, Michael Potts, Sanjive Qazi, Vuong Trieu

**Affiliations:** 1 Oncotelic Therapeutics, 29397 Agoura Road, Suite 107, Agoura Hills, CA 91301, USA; scott.myers@oncotelic.com (S.M.); michael.potts@oncotelic.com (M.P.); sanjive.qazi@sapubio.com (S.Q.); 2 Brush and Key Foundation, 4003 Jim Bowie, Agoura Hills, CA 91301, USA; shahdrashya27@gmail.com (D.S.); vtrieu3@autotelicinc.com (V.T.)

**Keywords:** hepatocellular carcinoma, pancreatic ductal adenocarcinoma, toll-like receptor, DNMT3A, GMPS

## Abstract

Hepatocellular carcinoma (HCC) and pancreatic ductal adenocarcinoma (PDAC) are highly lethal cancers marked by profound epigenetic and metabolic reprogramming. Among the candidate biomarkers, the DNA methyltransferase DNMT3A and the guanine monophosphate synthetase (GMPS) have emerged as potential prognostic drivers, yet their roles across tumor contexts remain unclear. Here, we demonstrate the application of KMplotter to interrogated pan-cancer transcriptomic and survival datasets encompassing over 7000 patients, complemented by expression profiling of normal, tumor, and metastatic tissues, and integrated tumor microenvironment (TME) analyses. Elevated DNMT3A and GMPS expression correlated with worse overall survival in HCC, particularly in Asian patients, while in PDAC, high DNMT3A but low GMPS expression predicted favorable outcomes. Both genes were consistently upregulated in tumors relative to normal tissues, with further increases in metastatic HCC. Immune deconvolution revealed that DNMT3A was linked to Th2/Treg-enriched niches, whereas GMPS overexpression coincided with high mutational burden or stromal enrichment, fostering immunosuppressive microenvironments. Comparative analysis of toll-like receptor signatures highlighted divergent antigen-sensing pathways, with HCC reflecting viral-driven immune exhaustion and PDAC showing self-antigen–associated signaling. Collectively, these findings position DNMT3A and GMPS as context-dependent biomarkers that integrate metabolic and immune cues to shape prognosis in liver and pancreatic cancer, offering mechanistic insight and translational relevance for patient stratification.

## 1. Introduction

Hepatocellular carcinoma (HCC), the predominant histologic type of primary liver cancer, represents an important global health burden. It ranks among the top three causes of cancer death worldwide, with a five-year survival rate of only 18% [1]. HCC accounts for 70–85% of all primary liver cancers and ranks as the second leading cause of cancer-related deaths in men [2]. The disease is frequently described as a leading cause of cancer mortality due to its progression being closely tied to disrupted metabolic processes, which complicates prognostic outcomes [3]. Biologically, HCC reflects a convergence of metabolic reprogramming and epigenetic dysregulation. Deregulated metabolism is a hallmark of cancer and circulating metabolite profiles have been linked to HCC [4]. Epigenetic abnormalities also play a critical role: genome-wide 5-methylcytosine levels are consistently reduced in HCC and correlate with higher tumor grade [5]. Epigenetic heterogeneity among tumor subsets likely contributes to poor prognosis, with hypomethylation persisting throughout tumor cell evolution [5]. Moreover, HCC demonstrates pronounced heterogeneity and complex clonal evolution, where both multicentric occurrence and intrahepatic metastasis can arise over time [6]. These biological complexities underscore the diagnostic and therapeutic challenges of the disease. Traditional clinicopathologic approaches have limited ability to predict outcomes [7]. In contrast, computational methods have identified promising biomarkers, including a four-driver gene signature with strong predictive accuracy [1] and a prognosis-related index based on seven key genes [7]. Network analyses have also highlighted GMPS as a recurrent hub gene in HCC [8]. Advances in early detection are underway: a metabolome-based liquid biopsy has successfully identified early-stage HCC and nominated RRM2, GMPS, BCAT1, PYCR2, and NEU1 as actionable candidates for prevention [4].

The epidemiology of HCC is closely linked to chronic viral hepatitis. Multiple large-scale studies have provided overwhelming evidence that chronic hepatitis B virus (HBV) infection plays a causal role in HCC development, with more than 50% of cases occurring in HBV carriers [9,10]. Viral hepatitis has been estimated in many studies to account for approximately 60–80% of HCC cases worldwide [11,12]. Hepatitis C virus (HCV) is also a major contributor [13]. The mechanisms connecting viral hepatitis to HCC are multifactorial, involving both direct oncogenic effects of the virus and indirect inflammation-driven pathways. HBV integration into the host genome promotes chromosomal instability through duplications, deletions, and translocations, and represents an early carcinogenic event [9,10]. Persistent expression of the viral regulatory HBV x protein further disrupts transcription, protein degradation, cell proliferation, and apoptotic signaling [9]. Unlike HCV, HBV can integrate into host DNA, providing an additional tumor-promoting mechanism [11]. Chronic hepatitis also accelerates the accumulation of genetic damage through immune-mediated inflammation and oxidative stress [10]. Beyond genetic instability, HBV exerts epigenetic effects by altering genomic methylation patterns and regulating microRNA expression [10]. Aberrant epigenetic regulation is a common feature of HCC [14]. The burden of liver cancer is particularly high in Asia, where HBV and HCV are prevalent [15]. In many countries, limited access to cancer control programs and early detection services exacerbates outcomes. This underscores the urgent need for simple, cost-effective screening tools, vaccination, and strategies to reduce risk factors [15]. Importantly, widespread vaccination against HBV and the availability of effective antiviral therapies are expected to significantly reduce the incidence of HCC [16].

Among emerging molecular candidates, the epigenetic writer DNMT3A and the metabolic enzyme GMPS have attracted attention for their potential prognostic significance in liver cancer. Evidence suggests that DNMT3A is upregulated early in hepatocarcinogenesis. mRNA levels of DNMT1 and DNMT3A are significantly higher in noncancerous livers affected by chronic hepatitis or cirrhosis compared with histologically normal livers, and these levels rise further in HCC [17]. This progressive increase parallels the accumulation of methylated tumor-suppressor genes observed from normal liver tissue to HCC [18], situating DNMT3A within a broader tumorigenic methylation program. Clinically, elevated DNMT3A expression has been linked to adverse outcomes: HCCs with more than a four-fold increase in DNMT3A mRNA display poorer recurrence-free survival [18]. In HCV-related cohorts, circulating DNMT3A mRNA has shown strong diagnostic performance in distinguishing HCC from cirrhosis, with an AUC of 0.958 at a defined cutoff, and with sensitivity and specificity values of 80.8% and 95.6%, respectively [19]. DNMT3A expression also correlates positively with alpha-fetoprotein levels and with multifocal disease [19]. Immunohistochemical studies further confirm its presence in high-grade dysplastic nodules and HCC lesions, reinforcing its role in early malignant transformation [20].

Mechanistic data supports DNMT3A as a driver of oncogenic behavior. Knockdown of DNMT3A reduces proliferation, suppresses colony formation, and demethylates the PTEN promoter, implicating direct silencing of tumor suppressors [21]. In addition, TGF-β–mediated enrichment of CD133^+^ liver cancer stem-like cells has been shown to rely on de novo DNA methyltransferases, and silencing these enzymes disrupts this process [22]. These findings highlight a reciprocal interaction between DNA methylation machinery and oncogenic signaling pathways, linking DNMT3A activity to both malignant progression and the maintenance of cancer stem-like features.

Parallel lines of evidence point to GMPS as another marker of poor prognosis in HCC. An integrated transcriptomic analysis identified a GMPS–RAMP3 gene pair as an independent predictor of survival with strong predictive power [23]. Functional studies provide mechanistic support: GMPS knockdown suppresses proliferation, promotes apoptosis, and sensitizes HCC cells to gemcitabine. Moreover, tumors with high GMPS/RAMP3 values show enrichment of cell-cycle-related pathways [23], connecting prognostic signals to actionable tumor cell vulnerabilities and suggesting translational potential.

Taken together, DNMT3A demonstrates multi-layered evidence—ranging from early overexpression in diseased liver to diagnostic accuracy in HCV-related cohorts, and from correlation with recurrence and survival to functional ties with cancer stemness—making it an actionable epigenetic biomarker in HCC [17,18,19]. However, heterogeneity across patient populations remains a challenge; some studies have failed to show linear relationships between DNMT expression and global methylation, highlighting the need for standardized assays and context-specific interpretation [17,24]. GMPS, while currently validated primarily within a gene-pair model, is supported by functional assays that underscore its role in aggressive tumor biology [23]. Broader external validation across diverse patient cohorts, etiologies, and treatment settings will be required before either candidate achieves clinical deployment. DNMT3A and GMPS have also been implicated in pancreatic tumor biology [25], motivating a cross-tumor investigation.

In this context, the present study aims to further explore DNMT3A and GMPS as prognostic and potentially predictive biomarkers in liver cancer and pancreatic cancer, with the goal of clarifying their biological roles and translational utility.

## 2. Results

### 2.1. DNMT3A and GMPS’ Impact on OS in Liver Cancer

In evaluating the patients in the km database of pan cancer, we found a significant impact on OS on liver hepatocellular carcinoma (HCC) and pancreatic ductal adenocarcinoma (PDAC). The database contains a total of 7489 patients, bladder carcinoma (*n* = 405), breast cancer (*n* = 1090), cervical squamous cell carcinoma (*n* = 304), esophageal adenocarcinoma (*n* = 80), esophageal squamous cell carcinoma (*n* = 81), head–neck squamous cell carcinoma (*n* = 500), kidney renal clear cell carcinoma (*n* = 530), kidney renal papillary cell carcinoma (*n* = 288), liver hepatocellular carcinoma (*n* = 371), lung adenocarcinoma (*n* = 513), lung squamous cell carcinoma (*n* = 501), ovarian cancer (*n* = 374), pancreatic ductal adenocarcinoma (*n* = 177), pheochromocytoma and paraganglioma (*n* = 178), rectum adenocarcinoma (*n* = 165), sarcoma (*n* = 259), stomach adenocarcinoma (*n* = 375), testicular germ cell tumor (*n* = 134), thymoma (*n* = 119), thyroid carcinoma (*n* = 502), and uterine corpus endometrial carcinoma (*n* = 543).

**Figure 1:**
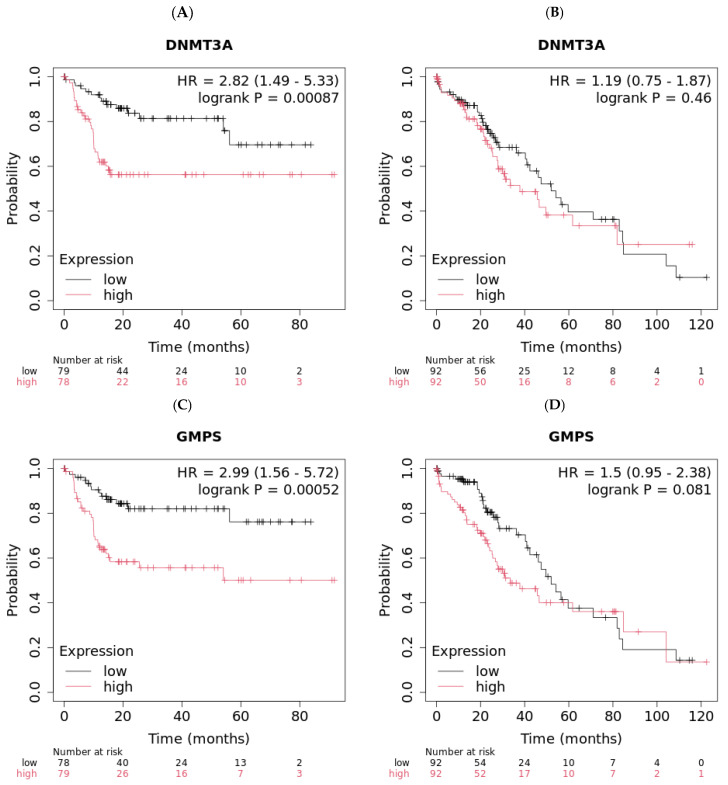
Low expression levels of DNMT3A and GMPS exhibited better overall survival (OS) in Asian liver cancer patients. (**A**) Asian liver cancer patients with low levels of DNMT3A exhibited improved OS compared to patients with high levels of DNMT3A (*n* = 157, log-rank *p* = 0.00087). (**B**) Caucasian liver cancer patients with different levels of DNMT3A exhibited similar OS (*n* = 184). (**C**) Asian liver cancer patients with low levels of GMPS exhibited improved OS compared to patients with high levels of GMPS (*n* = 157, log-rank *p* = 0.00052). (**D**) Caucasian liver cancer patients with different levels of GMPS exhibited similar OS (*n* = 184).

The demographic of HCC patients is shown in Table 1. High expression of either DNMT3A or GMPS is associated with worse OS only in the Asian population and not the Caucasian population (*p* = 0.00087 vs. not significant for DNMT3A, and *p* = 0.00052 vs. not significant for GMPS) (Figure 1). The Asian population with a low expression of DNMT3A showed better OS compared with the high group (upper quartile OS of 56.17 vs. 9.97 months, Figure 1A). The Caucasian population exhibited comparable median survival whether DNMT3A was low or high (52 vs. 37.83 months, Figure 1B). Similarly, the Asian population with low expression of GMPS showed better OS compared with the high group (upper quartile OS not reached, Figure 1C). The Caucasian population exhibited comparable median survival whether GMPS was low or high (52 vs. 33.5 months, Figure 1D). The impact of the tumor environment was evaluated in the same patient population against the expression of DNMT3A and GMPS (Supplementary Table S1). The DNMT3A effect exists in regulatory T cells of type 2 and T-helper cell-enriched environments with depleted basophils, B cells, and mesenchymal stem cells (MSC). The GMPS effect exists in high-mutational-burden environments with elevated type 1 T cells and macrophages and depleted basophils. We examined the patient population for risk factors for HCC. The incidence table is shown for those with greater than 5, and the primary differences between the Asian cohort and Caucasian cohort is the high incidence of hepatitis B in the Asian cohort (Supplementary Table S2). The disease stages in different races are also summarized (Supplementary Table S3).

### 2.2. DNMT3A and GMPS’ Impact on OS in Pancreatic Cancer

**Figure 2:**
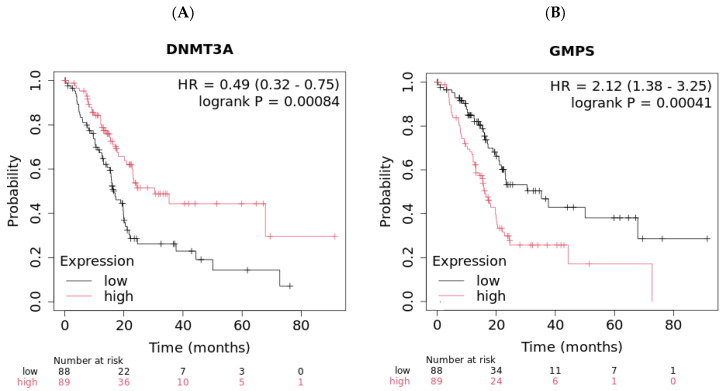
High expression levels of DNMT3A and low expression levels of GMPS exhibited better overall survival (OS) in pancreatic cancer patients (*n* = 177). (**A**) Pancreatic cancer patients with high levels of DNMT3A exhibited improved OS compared to patients with low levels of DNMT3A (log-rank *p* = 0.00084). (**B**) Pancreatic cancer patients with low levels of GMPS exhibited improved OS compared to patients with high levels of GMPS (log-rank *p* = 0.00041).

The demographic of this group of patients is shown in Table 2. High expression of these genes is associated with worse OS (*p* = 0.00084 for DNMT3A and *p* = 0.00041 for GMPS, Figure 2). Pancreatic cancer patients with high expression of DNMT3A showed better OS compared with the low group (30.43 vs. 16.6 months, Figure 2A). In contrast, pancreatic cancer patients with low expression of GMPS showed better OS compared with the high group (35.3 vs. 16.2 months, Figure 2B). The disease stages in different races were also summarized (Supplementary Table S3). The impact of tumor environment was evaluated in the same patient population against the expression of DNMT3A and GMPS (Supplementary Table S4). DNMT3A effect exists in environments depleted in type 1 T-helper cells. The GMPS effect exists in environments depleted in type 1 T-helper cells, CD4+ memory T cells, and macrophage-depleted environments with elevated MSC (Table 3).

When the tumor microenvironment (TME) signatures were compared, it was evidenced that the effects of DNMT3A and GMPS are similar between HCC and PDAC. In HCC, both DNMT3A and GMPS’ effects on OS are associated with a broad depletion of lymphoid subsets (basophils, B cells, CD4^+^/CD8^+^ T cells, eosinophils, MSC), while enriching macrophages, NK T cells, Tregs, Th1/Th2 cells, and maintaining high mutation burden. In PDAC, both the DNMT3A and GMPS effects on OS are associated with depleted basophils, B cells, CD4^+^/CD8^+^ T cells, and Th1 cells. However, unlike HCC, there are additional increases in eosinophils and MSC, and GMPS additionally depletes macrophages. The overall impact of the TME changes makes the HCC tumor overall hot but the immunologically skewed microenvironment and PDAC tumor overall cold, with a stromal-dominated immunosuppressed niche.

### 2.3. Expression Analysis Among Normal, Tumor, and Metastatic Tissues

**Figure 3:**
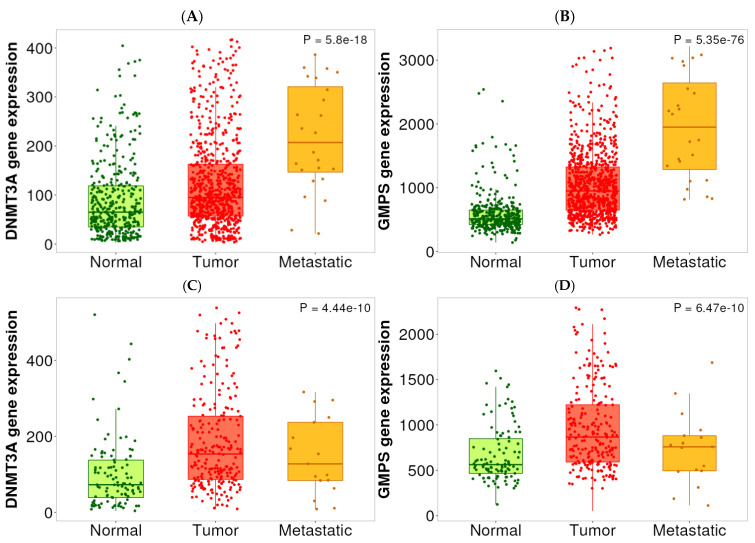
Expression levels of DNMT3A and GMPS in normal, HCC, and PDAC tissues. (**A**) The expression level of DNMT3A was elevated in HCC tissues compared with normal tissues. Metastatic lesions exhibited a further increase in DNMT3A expression in HCC tissues. Normal (*n* = 379), tumor (*n* = 806), and metastatic (*n* = 24). (**B**) The expression level of GMPS was elevated in HCC tissues compared to normal tissues. Metastatic lesions exhibited a further increase in GMPS expression in HCC tissues. Normal (*n* = 379), tumor (*n* = 806), and metastatic (*n* = 24). (**C**) The expression level of DNMT3A was elevated in PDAC tissues compared with normal tissues. Normal (*n* = 108), tumor (*n* = 248), and metastatic (*n* = 17). (**D**) The expression level of GMPS was elevated in PDAC tissues compared with normal tissues. Normal (*n* = 108), tumor (*n* = 248), and metastatic (*n* = 17).

As part of our selection of the targets for therapeutic intervention, we performed expression analysis to determine if there was elevated expression of DNMT3A and GMPS. As shown in Figure 3, DNMT3A and GMPS are overexpressed in tumor versus normal tissues of HCC (Figure 3A,B) and PDAC (Figure 3C,D). In metastatic lesions, they are further elevated in HCC, but not in PDAC (Figure 3A,B). A statistical summary is shown in Table 4.

### 2.4. Toll-like Receptors (TLRs) TME Analysis

Since HCC in Asians is caused by viral hepatitis, we examined the tumor signature for TLRs to determine the source of antigens in PDAC and HCC. As shown in Table 5, the TLRs TME signature was clearly suppressed for PDAC patients, but elevated in HCC patients, indicative of the chronic infection/immune exhaustion for PDAC and elevated immune response in HCC. Additionally, PDAC exhibited strong TLR2-driven self-sensing signatures, with other TLRs variably engaged and HCC characterized by other TLRs characteristic of non-self antigens. Because there is no other available database for HCC and the fact that the Caucasian population would serve as a natural control for the Asian population for Hepatitis B, we examined the TLR profiles for the Caucasian population. In this patient population, none of the relevant biomarkers have any prognostic significance (DNMT3A, GMPS, TLR1–TLR10). Surprisingly, statistical significance was achieved when GMPS were combined with TLRs. The data are shown in Table 5. Highlighted in bold are those with statistical significance. Most significant were TLR 2, 4, and 8.

We found another database within KMplotter using Affymetrix (Santa Clara, CA, USA) expression data across multiple cancer types, including PDAC (but not HCC). This entire database was examined for TLR1-10 profiles. The raw data is included in the Supplement Section (Supplementary Table S5). A summary of the data is shown in Table 6 as TLRs that are important either at increase above median expression or decrease below median expression. The data clearly shows the impact of TLRs on GMPS. Two of the tumors that requires elevated TLRs are gastric cancer and HCC in Asian populations; both are inflammatory tumors with chronic infection (HBV, *H. pylori*). All other tumors examined including PDAC are more reliant on low TLR expression: these include PDAC, AML, Lung, Ovarian, and, surprisingly, HCC in Caucasians, which was especially highly impacted by the TLRs. The former was dominated by TLR2 and the latter was dominated by TLR3 and TLR9, with each tumor indication exhibiting its own unique TLR profiles. DNMT3A was not utilized in this global analysis because its effect was not as robust nor as consistent as GMPS. When the Breast-TCGA database was analyzed, it was similar to the gastric cancer and HCC in Asians, with reliance on high TLR expression.

### 2.5. DNMT3A and GMPS Analyses at Protein Level

**Figure 4:**
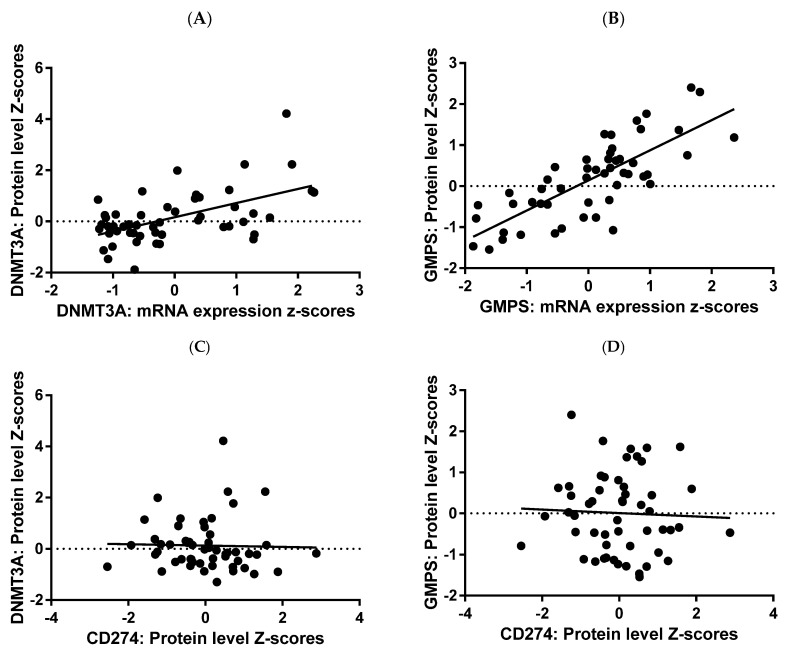
RNA protein correlation for GMPS and DNMT3A. RNA and protein expression data for GMPS and DNMT3A were plotted, demonstrating a strong correlation between RNA and protein in tumor tissues of breast cancer patients. Regression analysis yielded R^2^ value of 0.2926 and *p* value of <0.0001 for DNMT3A (**A**) and higher values of 0.5924 and *p* < 0.0001 for GMPS (**B**). There was no correlation between PDL1 (CD274) versus DNMT3A (**C**), nor GMPS (**D**) N = 71.

To validate the RNA expression data at the protein level, we examined all available public databases for data on both protein and RNA levels of DNMT3A and GMPS. Only one database exists: the breast proteogenomic study reported in Anurag M. et al. [26]. This is now part of the breast cancer database maintained at cbioportal.org. In this database, there was a strong correlation between mRNA expression and protein expression for both DNMT3A and GMPS (Figure 4A,B). There was no correlation between PDL1 (CD274) and DNMT3A, nor GMPS (Figure 4C,D).

**Figure 5:**
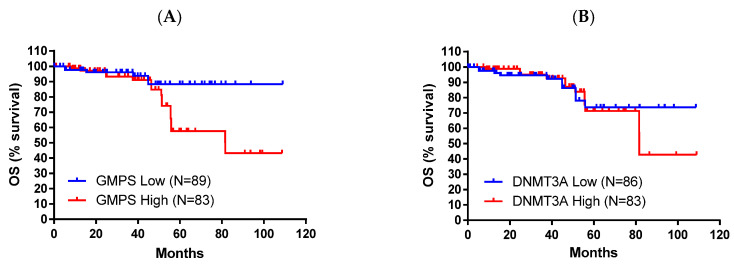
High protein levels of GMPS and DNMT3A exhibited worse overall survival (OS) in breast cancer patients. (**A**) Breast cancer patients with high levels of GMPS exhibited worse OS compared to patients with low levels of GMPS (log-rank *p* = 0.0247). (**B**) A similar trend was observed for DNMT3A, but did not achieve statistical significance.

All survival data for survival analysis were collected for breast cancer patients, available at cbioportal.org, with protein expression data for GMPS and DNMT3A. The protein data confirmed the RNA data, with high expressors having worse OS (Figure 5). 

## 3. Discussion

DNMT3A has a negative impact on OS in HCC, but a positive impact on OS in PDAC in our analysis. Despite that both show lymphoid depletion, but HCC is overall ‘hot’ and PDAC ‘cold/stromal’ by our signatures. In HCC, the DNMT3A low effect appears in a Th2/Treg-rich niche from which basophils, B cells, and mesenchymal stem cells (MSCs) are largely absent. In PDAC, the DNMT3A-high state coincides with loss of Th1 cells and only scarce inflammatory myeloid cells. DNMT3A uses S-adenosyl-methionine (SAM); sustained activity therefore forces a high SAM/SAH ratio and drives a hypermethylator program. In HCC, DNMT3A silencing increases IL-10 production in macrophages [27] and skews TAMs and MDSCs toward an M2-like immunosuppressive state [28]. IL-10/IL-4 signaling favors Th2 and FoxP3 + Treg expansion, but limits Th1 development. DNA and histone methylation suppress glycolytic and oxidative phosphorylation genes in myeloid cells. A low-glycolysis milieu suits FAO-dependent Tregs and Th2 cells, but it is hostile to basophils, B cells, and MSC, all of which rely on glucose. The same methylation program decreases MHC-I/co-stimulation, further curbing B cell help and basophil recruitment. DNMT3A therefore channels methionine-cycle flux into epigenetic marks that create an FAO-oriented, Th2/Treg-dominated environment while starving glycolysis-dependent basophils/B cells/MSC—exactly the constellation seen in HCC, and, when Th1 cells are also excluded, in PDAC.

We were unable to find a direct correlation between protein and mRNA. However, we did find a publication that indicates a direct correlation between protein and mRNA for DNMT3A in HCC. Park and colleagues measured DNMT3A in the same HCC samples at both the RNA and protein level and showed that the two are directly linked: DNMT3A mRNA was detectable in 59.3% of HCC, and DNMT3A protein immunostaining was seen in 48% of the same tumors. The DNMT3A mRNA expression profile showed significant correlation with its immunoreactivity (*p* = 0.022) [29]. Thus, in HCC, liver tumors’ higher DNMT3A transcript levels are significantly associated with higher DNMT3A protein expression. 

In HCC, GMPS over-expression appears in a TMB-high setting that still contains Type-1 (Th1/CD8) T cells and macrophages, but lacks basophils. In PDAC, GMPS-high lesions show loss of Th1, CD4 memory and macrophages, but an increase in MSC. GMPS is the rate-limiting step of de novo GMP synthesis. Its activity increased PD-L1 expression by regulating its ubiquitination and glycosylation modification and induced tumor immune evasion in HCC [30]. The pathway consumes glutamine/aspartate and generates GTP and purine catabolites. High GTP supports STT3A-mediated PD-L1 glycosylation, allowing for tumors with large neo-antigen burden to coexist with Th1 and macrophages while remaining protected. Adenosine produced from excess purines acts through A2A/A2B receptors to inhibit basophil chemotaxis and Th1 cytokine production; it also polarizes macrophages toward M2 or converts them into MDSCs, explaining macrophage loss in PDAC. Purine overflow nurtures stromal cells. In PDAC, CAF/MSC co-culture induced by GMPS over-expression increases tumor invasion [31]. GMPS-driven purine over-production fuels (i) rapid nucleotide demand, (ii) PD-L1 maturation, and (iii) adenosine-mediated immunosuppression. Where stroma is dense (PDAC), the same purines are scavenged by MSC/CAF, so macrophages and Th1 cells are diminished; where stroma is looser, (HCC) inflammatory cells still enter, but are functionally silenced by PD-L1 and adenosine. DNMT3A diverts methionine flux to SAM-dependent methylation, locking the niche into a low-glucose FAO-friendly state that selects for Tregs/Th2 cells and excludes glycolysis-dependent basophils/B cells/MSC. GMPS diverts glutamine/aspartate into purine synthesis, creating a PD-L1/adenosine shield. In immunogenic TMB-high HCC, this allows for Th1 and macrophages to be present but ineffective; in a CAF-rich PDAC matrix, the same purine excess is consumed by MSC/CAF, further reducing Th1/macrophage presence. Thus, the distinct immune landscapes of DNMT3A- and GMPS-driven tumors in liver versus pancreas can be rationalized by the different nutrient pathways that each enzyme controls and by organ-specific stromal contexts that dictate how those metabolites sculpt the tumor immune micro-environment.

When liver cells lose the ability to catabolizes SAM—either because glycine-N-methyl-transferase (GNMT) is deleted or other methyl-transferases such as DNMT3A acquire dominant activity—carbon from methionine is channeled into the transmethylation cycle instead of gluconeogenesis. In GNMT-knock-out mice, glucose precursor flux away from glucose formation, hepatic NAD^+^ falls and the NAD(P)H/NAD(P)^+^ ratio rises, resulting in reduced concentrations of gluconeogenic intermediates and an increase in pentose-phosphate- and glutathione-producing pathways [32]. The same study records lower blood glucose and reduction in liver glycogenolysis and gluconeogenesis, creating an extracellular environment poor in glucose but rich in methyl donors and anti-oxidants. Regulatory or Th2-skewed T cells, whose fitness relies on fatty-acid oxidation (FAO) and low glycolytic demand, thrive in such niches, whereas glycolysis-dependent basophils, B cells, or mesenchymal stromal cells are disfavored—a pattern repeatedly observed in Treg-dominated tumors [33,34]. DNMT3A mutations reinforce this setting by up-regulating NAMPT and accelerating the NAD salvage pathway, thereby fueling additional SAM-dependent methylation reactions and cell-cycle progression [35]. The combined effect is a low-glucose, FAO-friendly state that selects for Tregs/Th2 cells. In hepatocellular carcinoma and pancreatic ductal adenocarcinoma the amidotransferase GMPS couple’s nucleotide synthesis with immune escape. GMPS forms a bridge between nascent PD-L1 and the oligo-saccharyl-transferase catalytic sub-unit STT3A; this STT3A-dependent glycosylation stabilizes PD-L1 and impairs the tumor-killing function of CD8^+^ T cells [30]. The same purine bias enriches the TME with adenosine, a potent immunosuppressant. In pancreatic cancer, spatial transcriptomics show that high-purine (NT5E-high) regions lack response to immunotherapy and present an immunosuppressive microenvironment [31]. In an immunogenic TMB-high HCC, Th1 cells and macrophages are still recruited, but the GMPS-PD-L1/adenosine shield renders them ineffective. Angustmycin A, a GMPS inhibitor, significantly suppressed PD-L1 expression and increased the sensitivity to anti-CTLA-4 immunotherapy [30]. In the CAF-rich stroma of PDAC, purine metabolites are additionally scavenged by cancer-associated fibroblasts, further lowering nutrient availability and exacerbating the immunosuppression engendered by the progression of PC fibrosis [31,36]. DNMT3A/GNMT is associated with methionine carbon into SAM, drains gluconeogenic precursors, lowers glucose, favors FAO-positive regulatory subsets. GMPS is associated with glutamine/aspartate nitrogen into IMP/AMP/GMP, builds an adenosine-rich, PD-L1-shielded milieu. The liver is relatively glucose and amino acid rich and less desmoplastic, so methionine/SAM flux chiefly dictates immune composition, allowing for some inflammatory cells to persist but function poorly. The pancreas is intrinsically hypovascular and stroma-dense; excess purines are rapidly captured by CAFs and mesenchymal cells, intensifying nutrient competition and almost completely excluding Th1/macrophage effector cells. Taken together, the distinct immune landscapes of DNMT3A- and GMPS-driven tumors in liver versus pancreas can be rationalized by the different nutrient pathways that each enzyme controls and by organ-specific stromal contexts that dictate how those metabolites sculpt the tumor immune micro-environment.

Myeloid-adaptor signaling—almost always downstream of TLRs—is a central driver of the prototypical wound-healing/M2 milieu that characterizes PDAC. When primary human tumor-associated stroma (TAS) was incubated with pancreatic-cancer-conditioned medium, robust secretion of IL-6 and IL-8 ensued; the phenomenon was MyD88-dependent and directly suppressed CD4^+^ and CD8^+^ T cell proliferation while skewing the balance towards Th17 and away from Th1 cells [37]. In vivo, the same study documented an increased Th17:Th1 ratio in the blood of patients, underscoring the systemic reach of TLR/MyD88 signaling [37]. TLR7 is upregulated in both the epithelial and the stromal compartment of PDAC. Ligation of TLR7 in the K-ras mouse model vigorously accelerated tumor progression, interfaced with canonical NF-κB/MAPK cascades, induced STAT3 activation and caused loss of p16 and PTEN. Conversely, blockade of TLR7 protected against carcinogenesis [38]; hence, high TLR7 tone maintains an immunosuppressive tumor-permissive TME, whereas inhibition of that pathway delays tumor development.

Clinical correlative data mirror these experimental insights. In treatment-naïve metastatic PDAC high baseline plasma IL-6 and IL-10—two archetypal products of TLR → MyD88 → NF-κB signaling—were strongly associated with poorer overall survival (HR 1.16 and 1.28, respectively) [39]. A recent spatial epigenetic analysis showed that pancreatic tumor regions dominated by stroma harbor higher densities of tumor-associated macrophages while T cells are scarce [40]. Given that TAM accumulation is a recognized consequence of IL-6/IL-10–rich signaling, these findings further support the view that sustained TLR activity locks the PDAC TME in an M2/MDSC-dominated state and predicts shorter OS; low or blocked TLR activity breaks that loop and is associated with longer survival.

Roughly one quarter of HCCs belongs to an immune class that displays high expression levels of the CD274 molecule (PD-L1) and cytolytic genes together with fewer copy-number aberrations [41]. That class is enriched for transcripts induced by type-I/III interferons and Th1-skewed chemokines (CXCL9-11), a pattern typically downstream of virus-sensing TLR3/7/8/9. Patients with this immune-inflamed TLR-high phenotype have the best prognosis within the HCC spectrum [41]. By contrast, oncogenic cooperation between TLR4 and viral proteins can promote malignant progression. Ectopic TLR4 expression in hepatitis-C transgenic mice generated CD133^+^ tumor-initiating cells and mice with defective TGF-β signaling developed HCC in a TLR4-dependent manner [42]. Clinically, high serum TGF-β and expansion of TGF-β^+^ regulatory T cells are negatively correlated with overall survival of HCC patients [43], illustrating how TLR4→TGF-β crosstalk shapes an immunosuppressive TME and portends poor outcome. In keeping with these dualistic roles, the TCGA analysis identified two immune subclasses inside the immune group: an adaptive T cell response subtype with the best outcome and an exhausted T cell/TGF-β-high subtype with significantly worse survival [41]; hence, vigorous, TLR-driven antiviral/Th1 immunity favors tumor control, whereas a TLR-TGF-β axis that subverts immunity heralds shorter OS.

PDACs over-activate glycolysis generate an immunologically cold TME. A nine-gene metabolic-prognostic index (MPI) separates PAAD patients into high- and low-risk groups. High-MPI tumors are dominated by glycolysis and folate biosynthesis, show reduced CD8^+^ T cell infiltration and increased expression of immune checkpoints (PD-L1, TGF-β) and have significantly worse overall survival (OS) [36]. Tumor-derived exosomes signal through TLR2 on infiltrating macrophages, drive NF-κB-dependent aerobic glycolysis in these cells, and up-regulate PD-L1, thereby polarizing macrophages toward an immunosuppressive phenotype within a pre-metastatic niche [44]. Metabolic acidification and lactate accumulation reprogram M1-like macrophages to adopt a pro-cancerous fate and dampen cytotoxic T cell activity [45]. Collectively, high TLR2/NF-κB signaling, extensive glycolysis, and lactate build-up coincide with poor CD8^+^ T cell access and worse OS; conversely, tumors with lower glycolytic pressure (low MPI/low TLR2 signaling) display better immune infiltration and survival.

In HCC, lipid versus glycolysis heterogeneity produces mixed immune outcomes. Applying combined glycolysis and cholesterol biosynthesis signatures, 673 HCCs segregate into four metabolic subtypes: glycolytic (14.6%), mixed (10.4%), cholesterogenic (25.3%), and quiescent (49.8%) [46]. Glycolytic and mixed groups (subgroups including the glycolytic genotyping expression) have the highest mortality rate and are enriched for M0 macrophages; in these tumors, high M0 macrophage infiltration shortens OS, whereas abundant CD8^+^ T cells are scarce [46]. In contrast, the quiescent/cholesterogenic group shows low glycolytic stress; here, high CD8^+^ T cell and low M0-macrophage infiltration are associated with prolonged overall survival and patients with low naïve-B cell or low M0 macrophage density fare better [46]. Additional multi-omics studies confirm that the HCC subtype with the lowest metabolic alteration level (mHCC2) displays robust CD8^+^ T cell infiltration and the best outcome, whereas the highly reprogrammed mHCC1 subtype is strongly immunosuppressed [47].

**Figure 6:**
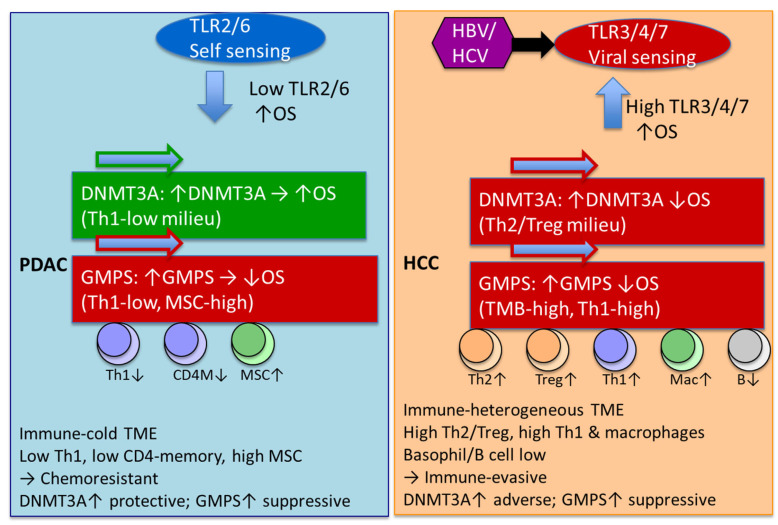
Divergent innate immune sensing and epigenetic drivers shape TME and survival in PDAC versus HCC. PDAC was predominantly TLR2/6 characteristic of self-antigens and low expression of TLR is associated with improved OS. HCC characterized by other TLRs characteristics of non-self-antigens and high expression is associated with improved OS. DNMT3A has a negative impact on OS in HCC, but a positive impact on OS in PDAC. In HCC, DNMT3A effect exists in regulatory T cells of type 2 and T-helper cell-enriched environments with depleted basophils, B cells, and MSC. In PDAC, DNMT3A effect exists in environments depleted in type 1 T-helper cells. GMPS effects are similar with HCC and PDAC has a negative impact on OS. In PDAC, the GMPS effect exists in environments depleted of type 1 T-helper cells, CD4^+^ memory T cells, and macrophage-depleted environments with elevated MSC. In HCC, the GMPS effect exists in high-mutational-burden environments with elevated type 1 T cells and macrophages and depleted basophils. Green arrow = favorable OS; red arrow = unfavorable OS; up/down arrow = higher/lower expression.

The data are summarized in Figure 6. The observed divergence in the prognostic impact of DNMT3A and GMPS between PDAC and HCC underscores the importance of tumor-specific immune and metabolic contexts in shaping clinical outcomes. In PDAC, the cold-immune TME, associated with reduced Th1 and CD4 memory cell infiltration but enriched for MSC stromal components, appears to favor a protective role for DNMT3A, while GMPS overexpression exacerbates immunosuppression and correlates with poor survival. This contrasts sharply with HCC, where an immune-heterogeneous TME characterized by elevated Th2, Treg, Th1, and macrophage infiltration, along with diminished B cell signatures, positions DNMT3A as an adverse prognostic factor. The deleterious impact of GMPS in HCC, particularly in association with high tumor mutational burden and Th1 activity, further highlights its contribution to immune evasion and poor patient outcomes. Moreover, the differential engagement of TLR pathways—TLR2-driven self-sensing signatures are prominent in PDAC and additional TLRs may contribute [44], self-sensing (TLR2/6) in PDAC versus viral-sensing (TLR3/4/7) in HCC—suggests that innate immune recognition mechanisms contribute to these distinct survival associations. Taken together, these findings indicate that DNMT3A and GMPS exert context-dependent effects that are closely linked to the TME and immunogenomic landscape of each tumor type, emphasizing their potential as biomarkers for patient stratification and precision immuno-metabolic therapies.

This study exploits the available KMplotter database to demonstrate novel TME differences between PDAC and HCC, especially the strong effect of TLR TME on both cancer types. It is anticipated that future analysis of other genes would point to a unifying relationship between TLR TME and cancer progression and survival. However, there are some notable limitations including that the survival analyses (KMplotter) and expression analyses (TNMplot v2) draw from overlapping but not identical patient cohorts. KMplotter integrates TCGA and GEO datasets primarily for survival outcome correlations, while TNMplot aggregates transcriptomic data from multiple platforms for expression-level comparisons across normal, tumor, and metastatic tissues. We acknowledge that using non-identical cohorts can in theory introduce (i) attenuation bias if strong tumor versus normal shifts coexist with modest intra-tumor variance and (ii) sampling bias because GTEx normal tissues derive from cancer-free donors. Analyses were kept orthogonal intentionally: TNMplot v2 was used solely for screening, and survival testing was restricted to the tumor subset within KMplotter. The complementary strength for KMplotter is survival-rich but normal-poor, whereas TNMplot v2 is normal-rich but survival-poor. By applying them orthogonally—to establish tumor-specific upregulation (TNMplot v2), and to test prognostic relevance in the nested tumor subset (KMplotter)—we maximized statistical power of the platforms. This study analyzes survival, gene expression, and TME. We are not aware of any other database with all three components. Due to limitation of this single database, additional preclinical and clinical studies will be conducted to validate the concepts described here, and the results are presented as hypothesis-generating only.

We did expand the analysis to a larger database within the KMplotter ecosystem using the TLR profile, which does not require deconvolution to determine TME profiles. The results confirmed and expanded our initial finding to breast, AML, gastric, lung, myeloma, and ovarian. The TLR expression profiles were used to quantify the TME environment instead of using the deconvolution method. This was deemed appropriate for the following reasons. Direct mirror of immune infiltration: Intratumoral CD3 and CD8 densities associated with a high TLR2 expression (*p* < 0.001 and *p* = 0.001) and a high TLR4 expression (*p* = 0.013 and *p* = 0.025). A low TLR5 immunoexpression associated with negative intratumoral CD3 (*p* = 0.001) and CD8 (*p* = 0.011) [48]. Thus, a single IHC stain for TLR2/4/5 on a colorectal carcinoma section largely recapitulates the manual counts of cytotoxic and helper T cells in both tumor nests and stroma. Links to functional immune status and prognosis: In human colonic disease, TLR-4 and IL-6 expression in the tumor microenvironment were associated with adenocarcinoma. Patients with very high levels of TLR-4 in the tumor stroma relapsed significantly earlier than those with lower expression levels [49]. In gastric cancers whose epithelial cells stain strongly for TLR5 show a 5-year disease-specific survival of 53% versus 38% for TLR5-low tumors (*p* = 0.014) [50]. Pancreatic ductal adenocarcinomas with high cytoplasmic TLR9 expression have a hazard ratio for cancer-specific death of 0.32 compared with TLR9-low cases (multivariate HR = 3.09 for low expression; *p* = 0.003) [51]. Mechanistic information about cytokine milieu- activation of tumor-cell TLRs drives the induction of inducible nitric oxide synthase (iNOS) and COX-2, which in turn increase TLR expression and promote a feed-forward loop, leading to tumor progression and the development of more aggressive tumor phenotypes [52]; hence, a strong TLR signal on pathology sections suggests an inflammatory, NF-κB-driven micro-environment rich in prostaglandins and NO. Therapy-guiding potential: TLR ligands are already approved (BCG, imiquimod) and >20 new agonists/antagonists are in clinical trials. As reviewed by Matijevic and Pavelic, TLRs have been used in numerous pre-clinical and clinical studies, especially combined with chemotherapy and radiotherapy [53].

The TLR profile separates out gastric cancer (GC) and HCC versus other cancer types. These two cancer types are unique in infection-related etiology. Gastric cancer (GC) is characterized by chronic *Helicobacter pylori* infection, which is the dominant carcinogenic driver; multi-omics profiling shows that *H. pylori*-positive GC harbors a distinct mutational/transcriptional and metabolic fingerprint compared with *H. pylori*-negative tumors [54]. *H. pylori* triggers a T-helper-1/IL-1β-rich milieu and induces PD-L1 on gastric epithelium, thereby shaping an inflamed but immunosuppressive micro-environment [55]. Gut dysbiosis beyond *H. pylori* (e.g., enrichment of *Collinsella* and *Ruminococcus* torques) further modulates cytokines and response to immunotherapy [54]. HCC in Asian population is characterized by ~80% arise on a background of chronic viral hepatitis (HBV or HCV) and/or cirrhosis-associated dysbiosis [41,56]. Viral antigens drive an immune-specific subclass with high PD-L1, cytolytic gene expression, and fewer chromosomal aberrations, whereas a second exhausted subclass is dominated by TGF-β signatures [41].

However, a limitation of this study is that the KMplotter and TNMplot analyses rely on bulk RNA-seq and microarray datasets, which cannot resolve the cellular origin of TLR transcripts within the tumor microenvironment. As a result, our findings with TLRs may reflect contributions from tumor cells, infiltrating immune cells, stromal fibroblasts, or other cell types. Because TLRs are expressed on diverse compartments, the observed associations between TLR levels and patient survival could arise from the combination of different biological processes depending on which cell types dominate the signal. Although we used TME deconvolution and cross-cohort comparisons to infer the biological contexts, further validations are still needed to confirm the hypothesis. Future studies incorporating scRNA-seq, multiplex imaging, or spatial transcriptomic analysis will be essential to determine whether the prognostic patterns identified are driven by tumor-intrinsic TLR signaling, immune-infiltrate composition, or stromal–immune interactions.

## 4. Materials and Methods

### 4.1. Domain-Specific Identification of PubMed Articles Augmented by Artificial Intelligence

PubMed searches using appropriate keywords (aging, cancer, DNMT3A, GMPS, liver cancer + methylation, metabolic reprogramming + cancer, methylation, pancreatic cancer, and aging) resulting in 32,264 abstracts were downloaded as text documents for processing using the Chatbot enabled tools developed at Oncotelic Therapeutics. Each abstract was then (aided using puppeteer 19.11.1), embedded, and transformed (langchain-openai 0.2.3, openai 1.52.0) into a vector of numbers capturing semantic similarity between text elements (tokens) and then stored in our Qdrant vector database (https://qdrant.tech/, accessed 25 March 2024). Semantically similar abstracts are transformed into the same vector “embedding” space, as the embedding has been trained to minimize the distance between pairs of abstracts in this space. Using an agglomerative clustering algorithm (hdbscan 0.8.39) to group the vectors, we automatically labeled these clusters using the question-answering model to identify any similarity between the abstracts corresponding to each cluster’s vectors. During the question-answering process, the user’s query is converted into an embedding vector. A similarity metric, such as cosine similarity, is then utilized to find the embedded abstract vectors nearest to the vector representing the query. The abstracts that correspond to these nearest vectors are subsequently provided to the question-answering model as context, alongside the original query, to produce a response to the user’s question.

React framework served as the backbone of the user interface, providing an open-source and flexible solution for developing powerful front-end user interfaces. (https://react.dev/, accessed 25 March 2024). In addition, we used the @mui/material (https://mui.com/, accessed on 25 March 2024) libraries for the interface’s design aspects and aimed to follow the material design guidelines closely. Serving the front end was Node.Js (https://nodejs.org/en, accessed on 25 March 2024). The Node.js libraries included in the project were @adobe/pdfservices-node-sdk 3.4.2@aws-sdk/client-s3 3.412.0, @langchain/community 0.2.5, @material-ui/core 4.12.4, @mui/base 5.0.0-beta.18, @mui/icons-material 5.11.16, @mui/material 5.15.20, @mui/styled-engine-sc 5.12.0, @mui/x-date-pickers 6.15.0, @qdrant/js-client-rest 1.4.0, carrot2 0.0.1, pdf2img 0.5.0, pdfjs-dist 4.5.136, puppeteer 19.11.1, react 18.2.0, sequelize 6.31.1, and zod 3.22.4.

The integration of our Chatbot technology applied to PubMed abstracts facilitated the rapid discovery of key primary publications used in the writing of this manuscript.

### 4.2. KMplotter and TNM Data

KMplotter database using the pan-cancer panel for investigating the effect of gene expression on OS as well as the TME (accessed June 2025). KMplotter is an online survival analysis tool, performing all calculations in real time. The background database is manually curated. Gene expression data and relapse free and overall survival information are downloaded from GEO, EGA, and TCGA. The database is handled by a PostgreSQL 12 server, which integrates gene expression and clinical data simultaneously. To analyze the prognostic value of a particular gene, the patient samples are split into two groups according to various quantile expressions of the proposed biomarker. The two patient cohorts are compared by a Kaplan–Meier survival plot, and the hazard ratio with 95% confidence intervals and log-rank *p*-value are calculated. Databases and clinical data are supervised and extended regularly [57]. Large, clinically annotated transcriptomic collections—spanning Affymetrix microarrays (primarily GEO cohorts) and RNA-seq (largely TCGA/ICGC)—have been assembled and harmonized in Győrffy’s ecosystem (KMplotter and companion databases) to enable survival-linked biomarker discovery across solid tumors. These frameworks standardize raw data, unify clinical endpoints, and support Cox/log-rank-based effect estimation and ranking; recent extensions cover breast, lung, ovarian, colon, pancreatic, and hepatocellular cohorts. Affymetrix microarrays quantify hybridization intensity for predefined probe sets (one or multiple probes per gene). Their strengths are lower cost, mature preprocessing, and broad legacy cohorts; their limitations include cross-hybridization, probe-sequence biases, compressed dynamic range, and difficulty resolving isoforms or novel transcripts. RNA-seq enumerates reads aligned to references, delivering wider dynamic range, detection of low-abundance genes, splice isoforms/fusions, and improved cross-study comparability when processed with uniform pipelines; challenges include count-based noise at low depth, library-composition effects, and batch variability [57,58,59,60,61,62].

The expression analyses was performed using TNMplot v2, which is an advanced web-based platform designed for transcriptomic analysis, integrating RNA-Seq and gene-chip data from 57 thousand samples. It facilitates differential gene expression analysis across normal, primary tumor, and metastatic tissues, supporting over 20 tumor types with publication-ready visualizations. The TNMplot v2 introduces stage-specific analysis, multi-gene analysis, correlation analysis tools, and expanded visualization features [63].

To estimate the relative abundance of immune and stromal components within each tumor sample, transcriptomic deconvolution was performed using a consensus framework integrating xCell, CIBERSORT (LM22 signature), and ESTIMATE algorithms. These algorithms were implemented within the KMplotter Pan-Cancer backend using harmonized RNA-seq expression matrices derived from TCGA cohorts (log_2_ TPM values, quantile-normalized and batch-corrected across studies). The CIBERSORT algorithm [64] employs ν-support vector regression to infer immune cell composition from bulk tumor expression data using the LM22 reference signature, which represents 22 leukocyte phenotypes (including B cell, T cell, NK, macrophage, and dendritic subsets). CIBERSORT defines immune populations using a supervised machine learning framework that deconvolves bulk gene-expression data into relative immune cell fractions based on a reference signature matrix. The reference matrix LM22 contains 547 marker genes characterizing 22 distinct human hematopoietic cell phenotypes spanning B cells, T cells, NK cells, monocytes/macrophages, dendritic cells, eosinophils, neutrophils, and mast cells. Each cell subset was defined from gene-expression profiles of purified leukocyte populations. Marker genes were selected by differential expression analysis followed by optimization to maximize between-subset discrimination (minimizing the condition number of the matrix). Genes that were also highly expressed in non-hematopoietic or tumor cells were removed using the Gene Enrichment Profiler and Cancer Cell Line Encyclopedia filters. The resulting LM22 matrix provides cell-type-specific expression fingerprints rather than relying solely on single surface markers, allowing for robust discrimination even among closely related lineages (e.g., resting vs. activated memory T cells). CIBERSORT estimates the abundance of each immune subset via ν-support vector regression (ν-SVR), a machine learning algorithm tolerant to noise and collinearity, providing accurate deconvolution from complex tumor transcriptomes. The extended platform, CIBERSORTx, introduces batch-effect correction (ComBat) to harmonize expression data across platforms and enables absolute quantification of immune fractions. Representative gene markers used to define the 22 immune populations include the following: B cells: MS4A1 (CD20), MZB1, XBP1; T cells: CD8A (CD8 T), CCR7 (naïve CD4), IL7R (memory resting CD4), IL2RA and FOXP3 (Tregs), CXCR5 and BCL6 (Tfh), TRDC (γδ T); NK cells: KLRB1, NKG7, IFNG, GZMB; Monocytes/macrophages: CD14, FCGR3A, IL1B, NOS2 (M1), CD163, MRC1 (M2); Dendritic cells: ITGAX, CD1C, CD83, CCR7; Mast cells/eosinophils/neutrophils: TPSAB1, TPSB2, CPA3, CLC, CEACAM8, S100A8/A9. These genes define transcriptional fingerprints of immune identity and activation state rather than relying on any single canonical surface antigen. The 2021 review by Le et al. confirms that among all of the digital cytometry approaches evaluated, CIBERSORT and CIBERSORTx B-mode consistently provide the highest correlation (r > 0.75) with flow-cytometry-derived cell fractions across multiple datasets, validating the LM22 gene set as a biologically meaningful framework for enumerating immune populations in bulk tumor RNA profiles [65].

## 5. Conclusions

This study demonstrates the power of the KMplotter platform in probing TME. KMplotter was used to establish DNMT3A and GMPS as TME dependent prognostic biomarkers with distinct implications in HCC and PDAC. Notably, in HCC, high expression of DNMT3A or GMPS was linked to poor OS, particularly in Asian patients, and was accompanied by immune heterogeneity driven by Th2/Treg enrichment, macrophage infiltration, and viral antigen-related TLR signaling. In contrast, in PDAC, DNMT3A overexpression correlated with improved OS within a cold-immune Th1-depleted milieu, whereas GMPS upregulation predicted poor OS in stromal-enriched immunosuppressive niches dominated by mesenchymal stem cells. Moreover, our hypothesis-generating findings suggest that divergent TLR TME signatures highlight fundamental differences in antigen sensing—self-antigen-driven pathways in PDAC versus viral-associated pathways in HCC—that shape tumor-immune interactions. This initial finding was expanded using the large Affymetrix database within KMplotter to Breast, AML, Gastric, Lung, Myeloma, and Ovarian.

## Figures and Tables

**Table 1:** Demographic table of hepatocellular carcinoma patients.

Hepatocellular Carcinoma			
		N	%
Total		370	100.00%
Gender	Male	249	67.30%
	Female	121	32.70%
Race	Caucasian	184	49.73%
	Asian	157	42.43%
	African	17	4.59%
Stage	1	171	46.22%
	2	85	22.97%
	3	85	22.97%
	4	5	1.35%
Grade	1	55	14.86%
	2	177	47.84%
	3	121	32.70%
	4	12	3.24%

**Table 2:** Demographic table of pancreatic cancer patients.

Pancreatic Ductal Adenocarcinoma			
		N	%
Total		177	100.00%
Gender	Male	97	54.80%
	Female	80	45.20%
Race	Caucasian	156	88.14%
	Asian	11	6.21%
	African	6	3.39%
Stage	1	21	11.86%
	2	146	82.49%
	3	3	1.69%
	4	4	2.26%
Grade	1	31	17.51%
	2	94	53.11%
	3	48	27.12%
	4	2	1.13%

**Table 3:** Tumor microenvironment (TME) signatures of both HCC and PDAC.

		HCC (*n* = 157)							PDAC (*n* = 177)					
		DNMT3A—Asian			GMPS—Asian				DNMT3A			GMPS		
		*p*-Value	OS Low	OS High	*p*-Value	OS Low	OS High		*p*-Value	OS Low	OS High	*p*-Value	OS Low	OS High
**All patients**		0.00087	*56.17*	*9.97*	0.00052	>57	12.5		0.00084	16.6	30.43	0.00041	35.3	16.2
**Basophils**	↓	**0.00081**	*56.17*	*9.97*	**0.00057**	>57	13.0	↓	0.0011	16.03	23.4	0.001	23.4	15.57
**B cells**	↓	**0.00054**	>57	10.5	0.0014	>57	13.0	↓	0.0029	15.77	24.6	0.0043	37.67	15.77
**CD4^+^ Tmem**	↓	0.0012	>57	12.5	0.0027	>57	12.5	↓	0.011	16.17	24.3	**0.00022**	37.67	15.53
**CD8^+^ T cells**	↓	0.0021	>57	12.5	0.0022	>57	13.0	↓	0.0032	15.57	23.17	0.0048	23.17	15.33
**Eosinophils**	↓	0.0012	*56.17*	*10*	0.001	>57	14.0	↑	0.0043	17.27	35.3	0.0039	35.3	17.27
**MSC**	↓	**0.00049**	>57	11.0	0.0015	>57	10.5	↑	0.014	16.6	23.4	**0.00068**	23.4	15.57
**Macrophages**	↑	0.0081	*56.17*	*9.97*	**0.00056**	>57	7.5	↓	0.045	8.13	13.1	**0.00079**	16.17	8.33
**NK T cells**	↑	0.0022	*54.07*	*9.3*	0.0026	*56.17*	*9.87*		n.s.	--	--	n.s.	--	--
**Reg T cells**	↑	**1.60 × 10^−5^**	>57	12.0	0.0014	>57	14.0		n.s.	--	--	n.s.	--	--
**Th1 cells**	↑	0.0047	*54.07*	*9.3*	**0.00038**	*56.17*	*9.3*	↓	**0.00085**	15.87	24.4	**0.00074**	30.43	15.77
**Th2 cells**	↑	**0.00055**	*25.6*	*5.7*	0.025	*15.63*	*6.5*		n.s.	--	--	n.s.	--	--
**Mutation Burden**	↑	0.0039	*54.07*	*8.73*	**5.8 × 10^−5^**	*56.17*	*8.73*		n.s.	--	--	n.s.	--	--

TME components able to maintain DNMT3A and the GMPS effect are shown. Bold highlighted are those where *p*-values are similar or less than that for all patients. The direction of expression is indicated by the direction of the arrow (up = above median, down = below median). The immunologic impact is indicated by color (red = hot tumor and green = cold tumor). Median OS in months are shown, italicized when only the upper quartile survival was available, and >57 as greater than the longest observed OS when the upper quartile survival was not reached. OS low = OS when the TME factor was below median; OS high = OS when the TME factor was above median. The prognostic effect of DNMT3A or GMPS (i.e., the separation of OS curves between high and low expressors) was most evident within specific immune microenvironment contexts, such as decreased B cells or CD8^+^ T cells or increased Tregs. The n.s. indicates no significant differences.

**Table 4:** Statistical summary of expression analysis in Figure 3.

	HCC (*n* = 157)		PDAC (*n* = 177)	
	DNMT3A	GMPS	DNMT3A	GMPS
Dunn.test.P				
Normal-Tumor	6.09 × 10^−16^	2.25 × 10^−69^	2.75 × 10^−11^	7.45 × 10^−11^
Normal-Metastatic	2.57 × 10^−8^	4.84 × 10^−22^	4.04 × 10^−2^	1.85 × 10^−1^
Tumor-Metastatic	8.79 × 10^−4^	4.23 × 10^−6^	1.16 × 10^−1^	2.21 × 10^−2^

**Table 5:** Toll-like receptor (TLR) TME signatures.

			PDAC (*n* = 177)			HCC—Asian (*n* = 157)			HCC—Caucasian (*n* = 184)		
			*p*-Value	OS Low	OS High	*p*-Value	OS Low	OS High	*p*-Value	OS Low	OS High
DNMT3A	TLR1	High	n.s.	--	--	n.s.	--	--	n.s.	--	--
		Low	0.0024	14.33	67.87	0.042	*54.07*	*11.47*	n.s.	--	--
	TLR2	High	n.s.	--	--	**7.9 × 10^−5^**	>57	6.0	n.s.	--	--
		Low	**0.00014**	15.33	67.87	n.s.	--	--	n.s.	--	--
	TLR3	high	0.0019	12.7	23.17	**0.00072**	>57	10.0	n.s.	--	--
		Low	n.s.	--	--	n.s.	--	--	n.s.	--	--
	TLR4	high	0.038	18.93	23.17	**0.00014**	>57	8.0	n.s.	--	--
		low	0.019	16.6	35.3	n.s.	--	--	n.s.	--	--
	TLR5	high	n.s.	--	--	0.0023	*56.17*	*5.7*	n.s.	--	--
		low	0.0015	15.67	67.87	n.s.	--	--	**0.0015**	59.7	31.03
	TLR6	high	n.s.	--	--	0.0081	>57	7.0	n.s.	--	--
		low	**0.00035**	15.77	72.73	n.s.	--	--	n.s.	--	--
	TLR7	high	n.s.	--	--	0.0055	*54.07*	*4.3*	n.s.	--	--
		Low	0.013	15.77	67.87	n.s.	--	--	n.s.	--	--
	TLR8	High	n.s.	--	--	**0.00033**	>57	5.0	n.s.	--	--
		Low	0.0045	15.87	67.87	n.s.	--	--	n.s.	--	--
	TLR9	High	n.s.	--	--	n.s.	--	--	n.s.	--	--
		Low	0.024	15.87	24.6	n.s.	--	--	n.s.	--	--
	TLR10	High	n.s.	--	--	0.0022	>57	5.0	n.s.	--	--
		Low	**0.00035**	15.67	67.87	n.s.	--	--	**0.036**	52.0	57.57
GMPS	TLR1	High	n.s.	--	--	n.s.	--	--	n.s.	--	--
		Low	**0.00024**	67.87	13.1	n.s.	--	--	**0.018**	71.03	27.57
	TLR2	High	n.s.	--	--	**0.00013**	>57	5.0	n.s.		
		Low	**0.000017**	67.87	15.33	n.s.	--	--	**0.0036**	71.03	29.97
	TLR3	high	n.s.	--	--	0.011	>57	15.0	n.s.	--	--
		Low	**0.00062**	67.87	19.93	0.037	*21.63*	*9.97*	n.s.	--	--
	TLR4	high	0.0093	23.17	18.17	**1.3 × 10^−5^**	>57	6.0	n.s.	--	--
		low	0.012	37.67	15.53	n.s.	--	--	**0.0077**	56.47	27.57
	TLR5	high	n.s.	--	--	0.0045	*56.17*	*6.5*	n.s.	--	--
		low	**0.00036**	37.67	13.13	0.044	>57	15.0	**0.036**	59.7	31.03
	TLR6	high	0.013	23.4	15.57	**0.00047**	>57	6.0	n.s.	--	--
		low	0.0077	17.03	9.77	n.s.	--	--	**0.016**	59.7	31.03
	TLR7	high	n.s.	--	--	0.0029	*54.07*	*4.67*	n.s.	--	--
		Low	0.0016	67.87	13.13	n.s.	--	--	**0.021**	59.7	27.57
	TLR8	High	n.s.	--	--	**0.00044**	>57	5.0	n.s.	--	--
		Low	0.0012	67.87	15.53	n.s.	--	--	**0.004**	59.7	29.97
	TLR9	High	n.s.	--	--	0.01	NA	NA	n.s.	--	--
		Low	0.032	30.43	15.87	n.s.	--	--	n.s.	--	--
	TLR10	High	n.s.	--	--	0.0048	>57	7.0	n.s.	--	--
		Low	0.034	37.67	14.33	0.012	NA	NA	n.s.	--	--

TLR impact on DNMT3A and the GMPS effect are shown. Median OS in months are shown, italicized when only the upper quartile survival was available, and >57 as greater than the longest observed OS when the upper quartile survival was not reached. OS low = OS when the TLR factor was below median; OS high = OS when the TME factor was above median. Highly significant *p*-values are shown in bold. The n.s. indicates no significant differences.

**Table 6:** TLR1-10 profile summary on GMPS in different cancers.

Low Expression	TLR1	TLR2	TLR3	TLR4	TLR5	TLR6	TLR7	TLR8	TLR9	TLR10	Sum
**PDAC**									1		1
**PDAC-Seq**	1	1	1		1						4
**Breast**	1										1
**Breast-TCGA**											0
**AML**			1		1		1		1		4
**Gastric**						1			1		2
**Lung**	1	1	1	1		1		1	1	1	8
**Myeloma**											0
**Ovarian**			1	1				1	1	1	5
**HCC-Seq**											0
**HCC-Seq-Caucasian**	1	1		1	1	1	1	1			7
**Total**	4	3	4	3	3	3	2	3	5	2	
**High Expression**	**TLR1**	**TLR2**	**TLR3**	**TLR4**	**TLR5**	**TLR6**	**TLR7**	**TLR8**	**TLR9**	**TLR10**	**Sum**
**PDAC**										1	1
**PDAC-Seq**											0
**Breast**											0
**Breast-TCGA**	1		1			1	1				4
**AML**											0
**Gastric**	1	1		1			1	1			5
**Lung**											0
**Myeloma**		1									1
**Ovarian**											0
**HCC-Seq-Asian**		1		1		1		1			4
**Total**	2	3	1	2	0	2	2	2	0	1	

Results of different TLRs on the GMPS effect are shown. Each positive impact is indicated as 1. Sum score = sum of all positive scores for each particular tumor indication. Total score = sum of all positive scores for each TLR.

## Data Availability

The original contributions presented in this study are included in the article/Supplementary Materials. Further inquiries can be directed to the corresponding author. We utilized the KMplotter database (https://kmplot.com/analysis/index.php?p=home, accessed on 30 June 2025) and TNMplot public database (https://tnmplot.com/analysis/, accessed on 30 June 2025).

## Supplementary Materials

The supporting information can be downloaded at https://www.mdpi.com/article/10.3390/ijms262411920/s1.

## References

[1] Guo H., Lu F., Lu R., Huang M., Li X., Yuan J., Wang F. (2023). A novel tumor 4-driver gene signature for the prognosis of hepatocellular carcinoma. Heliyon.

[2] Parpart S., Roessler S., Dong F., Rao V., Takai A., Ji J., Qin L.X., Ye Q.H., Jia H.L., Tang Z.Y. (2014). Modulation of miR-29 expression by alpha-fetoprotein is linked to the hepatocellular carcinoma epigenome. Hepatology.

[3] Zhu H., Xie Z. (2024). Therapeutic potential of tLyp-1-EV-shCTCF in inhibiting liver cancer stem cell self-renewal and immune escape via SALL3 modulation in hepatocellular carcinoma. Transl. Oncol..

[4] Schulze K., Rose T.D., Adlung L., Peschka M., Pagani F., Gorgulho J., Frundt T.W., Labgaa I., Haber P.K., Zimpel C. (2025). Metabolomic liquid biopsy dynamics predict early-stage HCC and actionable candidates of human hepatocarcinogenesis. JHEP Rep..

[5] Lin C.H., Hsieh S.Y., Sheen I.S., Lee W.C., Chen T.C., Shyu W.C., Liaw Y.F. (2001). Genome-wide hypomethylation in hepatocellular carcinogenesis. Cancer Res..

[6] Ohni S., Yamaguchi H., Hirotani Y., Nakanishi Y., Midorikawa Y., Sugitani M., Naruse H., Nakayama T., Makishima M., Esumi M. (2022). Direct molecular evidence for both multicentric and monoclonal carcinogenesis followed by transdifferentiation from hepatocellular carcinoma to cholangiocarcinoma in a case of metachronous liver cancer. Oncol. Lett..

[7] Deng L., Dou L., Huang X., Wang P., Shen N. (2025). Machine Learning-based Gene Biomarker Identification for Improving Prognosis and Therapy in Hepatocellular Carcinoma. Curr. Med. Chem..

[8] Zhang C., Peng L., Zhang Y., Liu Z., Li W., Chen S., Li G. (2017). The identification of key genes and pathways in hepatocellular carcinoma by bioinformatics analysis of high-throughput data. Med. Oncol..

[9] Tan Y.J. (2011). Hepatitis B virus infection and the risk of hepatocellular carcinoma. World J. Gastroenterol..

[10] Tarocchi M., Polvani S., Marroncini G., Galli A. (2014). Molecular mechanism of hepatitis B virus-induced hepatocarcinogenesis. World J. Gastroenterol..

[11] Tian Z., Xu C., Yang P., Lin Z., Wu W., Zhang W., Ding J., Ding R., Zhang X., Dou K. (2022). Molecular pathogenesis: Connections between viral hepatitis-induced and non-alcoholic steatohepatitis-induced hepatocellular carcinoma. Front. Immunol..

[12] El-Serag H.B. (2012). Epidemiology of viral hepatitis and hepatocellular carcinoma. Gastroenterology.

[13] Coleman W.B. (2003). Mechanisms of human hepatocarcinogenesis. Curr. Mol. Med..

[14] Ozen C., Yildiz G., Dagcan A.T., Cevik D., Ors A., Keles U., Topel H., Ozturk M. (2013). Genetics and epigenetics of liver cancer. N. Biotechnol..

[15] Pourhoseingholi M.A., Vahedi M., Baghestani A.R. (2015). Burden of gastrointestinal cancer in Asia; an overview. Gastroenterol. Hepatol. Bed Bench.

[16] Cha C., DeMatteo R.P. (2005). Molecular mechanisms in hepatocellular carcinoma development. Best Pract. Res. Clin. Gastroenterol..

[17] Saito Y., Kanai Y., Sakamoto M., Saito H., Ishii H., Hirohashi S. (2001). Expression of mRNA for DNA methyltransferases and methyl-CpG-binding proteins and DNA methylation status on CpG islands and pericentromeric satellite regions during human hepatocarcinogenesis. Hepatology.

[18] Oh B.K., Kim H., Park H.J., Shim Y.H., Choi J., Park C., Park Y.N. (2007). DNA methyltransferase expression and DNA methylation in human hepatocellular carcinoma and their clinicopathological correlation. Int. J. Mol. Med..

[19] Hassouna M.M., Naguib M., Radwan E.M., Abdel-Samiee M., Estaphan S., Abdelsameea E. (2020). DNA Methyltransferases as Potential Biomarkers for HCV Related Hepatocellular Carcinoma. Asian Pac. J. Cancer Prev..

[20] Choi M.S., Shim Y.H., Hwa J.Y., Lee S.K., Ro J.Y., Kim J.S., Yu E. (2003). Expression of DNA methyltransferases in multistep hepatocarcinogenesis. Hum. Pathol..

[21] Zhao Z., Wu Q., Cheng J., Qiu X., Zhang J., Fan H. (2010). Depletion of DNMT3A suppressed cell proliferation and restored PTEN in hepatocellular carcinoma cell. J. Biomed. Biotechnol..

[22] Hu S., Luo X., Qian J., Hou Y., Shi W. (2023). High expression of DNMT3A and DNMT3B regulatory factors of TGFB in non-neoplastic liver tissues of HCC. Cell. Mol. Biol..

[23] Chen Z., Zeng Y., Ma P., Xu Q., Zeng L., Song X., Yu F. (2025). Integrated GMPS and RAMP3 as a signature to predict prognosis and immune heterogeneity in hepatocellular carcinoma. Gene.

[24] Siddiqui N.N., Ul Haq A., Siddiqui O.A., Khan R. (2016). DNA methyltransferase 1, 3a, and 3b expression in hepatitis C associated human hepatocellular carcinoma and their clinicopathological association. Tumour Biol..

[25] Jing W., Song N., Liu Y.P., Qu X.J., Qi Y.F., Li C., Hou K.Z., Che X.F., Yang X.H. (2019). DNMT3a promotes proliferation by activating the STAT3 signaling pathway and depressing apoptosis in pancreatic cancer. Cancer Manag. Res..

[26] Anurag M., Jaehnig E.J., Krug K., Lei J.T., Bergstrom E.J., Kim B.J., Vashist T.D., Huynh A.M.T., Dou Y., Gou X. (2022). Proteogenomic Markers of Chemotherapy Resistance and Response in Triple-Negative Breast Cancer. Cancer Discov..

[27] Mba Medie F., Sharma-Kuinkel B.K., Ruffin F., Chan L.C., Rossetti M., Chang Y.L., Park L.P., Bayer A.S., Filler S.G., Ahn R. (2019). Genetic variation of DNA methyltransferase-3A contributes to protection against persistent MRSA bacteremia in patients. Proc. Natl. Acad. Sci. USA.

[28] Zhang M., Pan X., Fujiwara K., Jurcak N., Muth S., Zhou J., Xiao Q., Li A., Che X., Li Z. (2021). Pancreatic cancer cells render tumor-associated macrophages metabolically reprogrammed by a GARP and DNA methylation-mediated mechanism. Signal Transduct. Target. Ther..

[29] Park H.J., Yu E., Shim Y.H. (2006). DNA methyltransferase expression and DNA hypermethylation in human hepatocellular carcinoma. Cancer Lett..

[30] Guo X., Cui T., Sun L., Fu Y., Cheng C., Wu C., Zhu Y., Liang S., Liu Y., Zhou S. (2025). A STT3A-dependent PD-L1 glycosylation modification mediated by GMPS drives tumor immune evasion in hepatocellular carcinoma. Cell Death Differ..

[31] Zhang J., Zhang X., Wu R., Dong C.S. (2025). Unveiling purine metabolism dysregulation orchestrated immunosuppression in advanced pancreatic cancer and concentrating on the central role of NT5E. Front. Immunol..

[32] Hughey C.C., James F.D., Wang Z., Goelzer M., Wasserman D.H. (2019). Dysregulated transmethylation leading to hepatocellular carcinoma compromises redox homeostasis and glucose formation. Mol. Metab..

[33] Wang Y.A., Li X.L., Mo Y.Z., Fan C.M., Tang L., Xiong F., Guo C., Xiang B., Zhou M., Ma J. (2018). Effects of tumor metabolic microenvironment on regulatory T cells. Mol. Cancer.

[34] Yan Y., Huang L., Liu Y., Yi M., Chu Q., Jiao D., Wu K. (2022). Metabolic profiles of regulatory T cells and their adaptations to the tumor microenvironment: Implications for antitumor immunity. J. Hematol. Oncol..

[35] Yang X., Wang X., Yang Y., Li Z., Chen Y., Shang S., Wang Y. (2023). DNMT3A mutation promotes leukemia development through NAM-NAD metabolic reprogramming. J. Transl. Med..

[36] Song W., Yu Y., Wang S., Cui Z., Zhu Q., Liu W., Wei S., Chi J. (2025). Metabolic reprogramming shapes the immune microenvironment in pancreatic adenocarcinoma: Prognostic implications and therapeutic targets. Front. Immunol..

[37] Delitto D., Delitto A.E., DiVita B.B., Pham K., Han S., Hartlage E.R., Newby B.N., Gerber M.H., Behrns K.E., Moldawer L.L. (2017). Human Pancreatic Cancer Cells Induce a MyD88-Dependent Stromal Response to Promote a Tumor-Tolerant Immune Microenvironment. Cancer Res..

[38] Ochi A., Graffeo C.S., Zambirinis C.P., Rehman A., Hackman M., Fallon N., Barilla R.M., Henning J.R., Jamal M., Rao R. (2012). Toll-like receptor 7 regulates pancreatic carcinogenesis in mice and humans. J. Clin. Investig..

[39] Farren M.R., Mace T.A., Geyer S., Mikhail S., Wu C., Ciombor K., Tahiri S., Ahn D., Noonan A.M., Villalona-Calero M. (2016). Systemic Immune Activity Predicts Overall Survival in Treatment-Naive Patients with Metastatic Pancreatic Cancer. Clin. Cancer Res..

[40] Tomasich E., Muhlbacher J., Woran K., Hatziioannou T., Herac M., Kleinberger M., Berger J.M., Dibon L.K., Berchtold L., Heller G. (2024). Immune cell distribution and DNA methylation signatures differ between tumor and stroma enriched compartment in pancreatic ductal adenocarcinoma. Transl. Res..

[41] Sia D., Jiao Y., Martinez-Quetglas I., Kuchuk O., Villacorta-Martin C., Castro de Moura M., Putra J., Camprecios G., Bassaganyas L., Akers N. (2017). Identification of an Immune-specific Class of Hepatocellular Carcinoma, Based on Molecular Features. Gastroenterology.

[42] Machida K., Feldman D.E., Tsukamoto H. (2015). TLR4-dependent tumor-initiating stem cell-like cells (TICs) in alcohol-associated hepatocellular carcinogenesis. Biological Basis of Alcohol-Induced Cancer.

[43] An Y., Gao S., Zhao W.C., Qiu B.A., Xia N.X., Zhang P.J., Fan Z.P. (2018). Transforming growth factor-beta and peripheral regulatory cells are negatively correlated with the overall survival of hepatocellular carcinoma. World J. Gastroenterol..

[44] Morrissey S.M., Zhang F., Ding C., Montoya-Durango D.E., Hu X., Yang C., Wang Z., Yuan F., Fox M., Zhang H.G. (2021). Tumor-derived exosomes drive immunosuppressive macrophages in a pre-metastatic niche through glycolytic dominant metabolic reprogramming. Cell Metab..

[45] Jin X., Zhang N., Yan T., Wei J., Hao L., Sun C., Zhao H., Jiang S. (2025). Lactate-mediated metabolic reprogramming of tumor-associated macrophages: Implications for tumor progression and therapeutic potential. Front. Immunol..

[46] Wei X., Michelakos T., He Q., Wang X., Chen Y., Kontos F., Wang H., Liu X., Liu H., Zheng W. (2023). Association of Tumor Cell Metabolic Subtype and Immune Response with the Clinical Course of Hepatocellular Carcinoma. Oncologist.

[47] Yang J., Zeng L., Chen R., Zheng S., Zhou Y., Chen R. (2023). Characterization of heterogeneous metabolism in hepatocellular carcinoma identifies new therapeutic target and treatment strategy. Front. Immunol..

[48] Beilmann-Lehtonen I., Kasurinen J., Hagstrom J., Kaprio T., Bockelman C., Haglund C. (2023). High tissue expression of TLRs combined with high density of tumor infiltrating lymphocytes predicts a better prognosis in colorectal cancer patients. PLoS ONE.

[49] Cammarota R., Bertolini V., Pennesi G., Bucci E.O., Gottardi O., Garlanda C., Laghi L., Barberis M.C., Sessa F., Noonan D.M. (2010). The tumor microenvironment of colorectal cancer: Stromal TLR-4 expression as a potential prognostic marker. J. Transl. Med..

[50] Kasurinen A., Hagstrom J., Laitinen A., Kokkola A., Bockelman C., Haglund C. (2019). Evaluation of toll-like receptors as prognostic biomarkers in gastric cancer: High tissue TLR5 predicts a better outcome. Sci. Rep..

[51] Leppanen J., Helminen O., Huhta H., Kauppila J.H., Isohookana J., Haapasaari K.M., Lehenkari P., Saarnio J., Karttunen T.J. (2017). High toll-like receptor (TLR) 9 expression is associated with better prognosis in surgically treated pancreatic cancer patients. Virchows Arch..

[52] Ridnour L.A., Cheng R.Y., Switzer C.H., Heinecke J.L., Ambs S., Glynn S., Young H.A., Trinchieri G., Wink D.A. (2013). Molecular pathways: Toll-like receptors in the tumor microenvironment--poor prognosis or new therapeutic opportunity. Clin. Cancer Res..

[53] Matijevic T., Pavelic J. (2010). Toll-like receptors: Cost or benefit for cancer?. Curr. Pharm. Des..

[54] Shang F., Cao Y., Wan L., Ren Z., Wang X., Huang M., Guo Y. (2023). Comparison of Helicobacter pylori positive and negative gastric cancer via multi-omics analysis. mBio.

[55] Bednarz-Misa I., Fortuna P., Diakowska D., Jamrozik N., Krzystek-Korpacka M. (2020). Distinct Local and Systemic Molecular Signatures in the Esophageal and Gastric Cancers: Possible Therapy Targets and Biomarkers for Gastric Cancer. Int. J. Mol. Sci..

[56] Greten T.F., Schwabe R., Bardeesy N., Ma L., Goyal L., Kelley R.K., Wang X.W. (2023). Immunology and immunotherapy of cholangiocarcinoma. Nat. Rev. Gastroenterol. Hepatol..

[57] Gyorffy B. (2024). Integrated analysis of public datasets for the discovery and validation of survival-associated genes in solid tumors. Innovation.

[58] Posta M., Gyorffy B. (2025). Pathway-level mutational signatures predict breast cancer outcomes and reveal therapeutic targets. Br. J. Pharmacol..

[59] Gyorffy B. (2023). Discovery and ranking of the most robust prognostic biomarkers in serous ovarian cancer. GeroScience.

[60] Gyorffy B. (2024). Transcriptome-level discovery of survival-associated biomarkers and therapy targets in non-small-cell lung cancer. Br. J. Pharmacol..

[61] Menyhart O., Nagy A., Gyorffy B. (2018). Determining consistent prognostic biomarkers of overall survival and vascular invasion in hepatocellular carcinoma. R. Soc. Open Sci..

[62] Posta M., Gyorffy B. (2023). Analysis of a large cohort of pancreatic cancer transcriptomic profiles to reveal the strongest prognostic factors. Clin. Transl. Sci..

[63] Bartha A., Gyorffy B. (2021). TNMplot.com: A Web Tool for the Comparison of Gene Expression in Normal, Tumor and Metastatic Tissues. Int. J. Mol. Sci..

[64] Newman A.M., Liu C.L., Green M.R., Gentles A.J., Feng W., Xu Y., Hoang C.D., Diehn M., Alizadeh A.A. (2015). Robust enumeration of cell subsets with tissue expression profiles. Nat. Methods.

[65] Le T., Aronow R.A., Kirshtein A., Shahriyari L. (2021). A review of digital cytometry methods: Estimating the relative abundance of cell types in a bulk of cells. Brief. Bioinform..

