# Supplementary material for: Comparative Tumor Microenvironment Analysis for HCC and PDAC Using KMplotter
Source: Int J Mol Sci. 2025 Dec 10;26(24):11920. doi: 10.3390/ijms262411920 (PMC12732809; doi:10.3390/ijms262411920)
Supplement: Supplementary file 1 [file ijms-26-11920-s001.zip › ijms-3939715-supplementary.pdf]

Supplement material

Table 1. Tumor microenvironment analyses for Asian liver cancer patients. Bold indicates greater statistical significance than the entire population (0.00087 for DNMT3A and 0.00052 for GMPS). NA = Not available; ns = not significant.

	**DNMT3A- Asian**			**GMPS - Asian**		
	**p-value**	**OS low**	**OS high**	**p-value**	**OS low**	**OS high**
All Asian patients	0.00087	56.17	9.97	0.00052	NA	NA
Basophilis-deceased	**0.00081**	56.17	9.97	**0.00057**	NA	NA
B cells : enriched	0.032	56.17	7.23	ns	56.17	5.7
B cells- deceased	**0.00054**	NA	NA	**0.0014**	NA	NA
CD4+ memory T- cells - enriched	ns	56.17	7.23	ns	56.17	5.7
CD4+ memory T-cells - decreased	**0.0012**	NA	NA	**0.0027**	NA	NA
CD8+ T-cells - enriched	ns	56.17	7.23	ns	56.17	5.7
CD8+ T-cells- decreased	**0.0021**	NA	NA	**0.0022**	NA	NA
Eosinophils- decreased	**0.0012**	56.17	10	**0.001**	NA	NA
Macrophages- enriched	**0.0081**	56.17	9.97	**0.00056**	NA	NA
Macrophages- deceased	0.026	54.07	9.87	ns	NA	NA
Mesenchymal stem cells- enriched	ns	21.63	9.97	ns	54.07	10.13
Mesenchymal stem cells- deceased	**0.00049**	NA	NA	**0.0015**	NA	NA
Natural killer T cells- enriched	**0.0022**	54.07	9.3	**0.0026**	56.17	9.87
Natural killer T cells- deceased	ns	NA	NA	ns	NA	NA
Regulatory T-cells- enriched	**1.60E-05**	NA	NA	**0.0014**	NA	NA
Regulatory T-cells- deceased	ns	17.83	10	ns	17.83	9.97
Type 1 T-helper cells- enriched	**0.0047**	54.07	9.3	**0.00038**	56.17	9.3
Type 1 T-helper cells- deceased	0.013	NA	NA	ns	NA	NA
Type 2 T-helper cells- enriched	**0.00055**	25.6	5.7	0.025	15.63	6.5
Type 2 T-helper cells- deceased	0.045	NA	NA	ns	NA	NA
Mutation high	**0.0039**	54.07	8.73	**5.8e-5**	56.17	8.73
Mutation low	ns	NA	NA	ns	NA	NA

Table 2. Incidence table for Asian and Caucasian patient populations.

History hepato carcinoma risk factor	(A) ASIAN (N=161)	(B) WHITE (N=187)
**Hepatitis B**	72	5
**No History of Primary Risk Factors**	25	63
**Alcohol consumption**	25	41
**Hepatitis C**	5	18
**Alcohol consumption|Hepatitis B**	18	1
**Alcohol consumption|Hepatitis C**	1	11
**Non-Alcoholic Fatty Liver Disease**	1	9
**Hemochromatosis**	0	6

Table 3. Disease stages in different races.

HCC	Asian median OS (months)					
**Stage**	Low DNMT3A	High DNMT3A	P value	Low GMPS	High GMPS	P value
**All**	56.17	9.97	0.0009	NA	NA	0.0005
**1**	NA	NA	0.675	NA	NA	0.2909
**2**	NA	NA	0.0408	NA	NA	0.0936
**3**	54.07	10.1	0.0562	9.27	4.67	0.0237
**4**	NA	NA	NA	NA	NA	NA

HCC	White median OS (months)					
**Stage**	Low DNMT3A	High DNMT3A	P value	Low GMPS	High GMPS	P value
**All**	52	37.83	0.4552	52	33.5	0.0813
**1**	56.47	45.73	0.9876	71.03	45.73	0.0488
**2**	46.2	61.73	0.7425	108.6	61.73	0.7615
**3**	59.7	33.5	0.1362	49.67	33.5	0.7698
**4**	NA	NA	NA	NA	NA	NA

PDAC	All patient median OS (months)					
**Stage**	Low DNMT3A	High DNMT3A	P value	Low GMPS	High GMPS	P value
**All**	16.6	30.43	0.0008	35.3	16.2	0.0004
**1**	NA	NA	0.2056	NA	NA	0.0526
**2**	16.03	23.17	0.0085	20.03	15.87	0.0209
**3**	NA	NA	NA	NA	NA	NA
**4**	NA	NA	NA	NA	NA	NA

Table 4. Tumor Microenvironment Analyses for pancreatic cancer patients. Highlighted cells are those maintaining high *p* value relative that of all patients.

	DNMT3A- all pts			GMPS- all pts		
	p value	OS low	OS high	p value	OS low	OS high
All patients	0.00084	16.6	30.43	0.00041	35.3	16.2
Basophilis-deceased	**0.0011**	16.03	23.4	**0.001**	23.4	15.57
B cells : enriched	ns	19.87	23.07	0.034	30.43	16.6
B cells- deceased	**0.0029**	15.77	24.6	**0.0043**	37.67	15.77
CD4+ memory T- cells - enriched	ns	18.93	30.43	ns	19.87	19.73
CD4+ memory T-cells - decreased	**0.011**	16.17	24.3	**0.00022**	37.67	15.53
CD8+ T-cells - enriched	ns	30.43	23.4	ns	35.3	20.1
CD8+ T-cells- decreased	**0.0032**	15.57	23.17	**0.0048**	23.17	15.33
eosinophils- enriched	**0.0043**	17.27	35.3	**0.0039**	35.3	17.27
eosinophils- decreased	ns	12.6	16.17	ns	15.57	13.13
macrophages- enriched	0.0068	16.6	30.43	ns	23.03	16.6
macrophages- deceased	**0.045**	8.13	13.1	**0.00079**	16.17	8.33
Mesenchymal stem cells- enriched	**0.014**	16.6	23.4	**0.00068**	23.4	15.57
Mesenchymal stem cells- deceased	ns	16.17	16.2	ns	50.07	16.2
natural killer T cells- enriched	ns	15.77	24.6	0.025	35.3	13.13
natural killer T cells- deceased	0.026	19.73	30.43	0.016	30.43	17.27
regulatory T-cells- enriched	0.035	19.73	30.43	0.024	37.67	17.27
regulatory T-cells- deceased	ns	17.03	23.4	0.011	23.4	15.87
type 1 T-helper cells- deceased	**0.00085**	15.87	24.4	**0.00074**	30.43	15.77
type 2 t-helper cells- enriched	0.0031	11.13	24.6	ns	17.73	11.13
type 2 t-helper cells- deceased	ns	20.1	23.4	0.0092	37.67	19.93
Mutation high	0.0063	15.33	72.73	ns	23.03	17.27
Mutation low	0.025	17.03	23.4	0.037	35.3	19.73

Table 5. TLR1-10 profile from Affymetrix expression data across multiple cancers.

	PDAC		p value	OS Low	OS High
**GMPS**	TLR1	high	7.00E-04	20.17	15.78
		low	0.012	20.9	16.07
	TLR2	high	ns	19.5	15
		low	ns	21.09	17.43
	**TLR3**	high	ns	20.27	18
		low	0.00068	20.33	15.33
	TLR4	high	0.0017	20.13	15.2
		low	0.024	20.7	17
	TLR5	high	0.011	20.9	17.17
		low	0.0095	20.1	15.7
	TLR6	high	0.0096	19.3	15.87
		low	0.011	20.7	17
	**TLR7**	high	0.0016	21.77	16.03
		low	0.14	19.54	16.07
	**TLR8**	high	0.00066	23.17	16.17
		low	0.0055	20.2	15
	**TLR9**	high	0.043	19.8	16
		low	0.00015	21.37	15.73
	TLR10	high	0.00013	22.27	16
		low	ns	19.73	17.03
	**Breast**	**upper quartile**			
	TLR1	high	ns	78	75.37
		low	0.00066	136.8	56.28
	TLR2	high	0.0029	179.01	205.64
		low	0.00065	125.92	67.08
	**TLR3**	high	0.0094	169.2	81.87
		low	ns	79.2	63.25
	TLR4	high	0.0097	136.8	68.4
		low	ns	75.83	56.4
	TLR5	high	0.0031	169.2	198.44
		low	0.00072	135.84	68.04
	TLR6	high	ns	123.6	81.87
		low	0.0056	106.8	56.4
	**TLR7**	high	0.012	191.21	205.64
		low	3.60E-05	135.84	59.28
	**TLR8**	high	ns	NA	NA
		low	0.00014	106.8	44.6
	**TLR9**	high	ns	84	67.4
		low	0.0057	135.84	59.76

TLR10	high	ns	136.8	138
	low	ns	63.36	44.4
**AML**				
TLR1	high	ns	16.7	14.1
	low	ns	16.3	11.8
TLR2	high	ns	18	14.8
	low	ns	15.2	12.1
**TLR3**	high	ns	15	11.3
	low	0.012	19.2	13.4
TLR4	high	ns	19.2	16.1
	low	ns	18	20.2
TLR5	high	ns	16.6	13.5
	low	0.0035	19.8	11.3
TLR6	high	ns	19.2	19.6
	low	ns	17.7	15.7
**TLR7**	high	ns	13.9	15.7
	low	0.0022	20.1	11.3
**TLR8**	high	ns	16.7	14.8
	low	ns	23.3	18
**TLR9**	high	ns	17.7	14.9
	low	0.0035	30.3	15.7
TLR10	high	ns	17.7	19.9
	low	ns	19.2	17.3
**Colon**				
TLR1	high	ns		
	low	ns		
TLR2	high	ns		
	low	ns		
**TLR3**	high	ns		
	low	ns		
TLR4	high	ns		
	low	ns		
TLR5	high	ns		
	low	ns		
TLR6	high	ns		
	low	ns		
**TLR7**	high	ns		
	low	ns		
**TLR8**	high	ns		
	low	ns		
**TLR9**	high	ns		

		low	ns		
	TLR10	high	ns		
		low	ns		
	**DLBCL**				
	TLR1	high	ns		
		low	ns		
	TLR2	high	ns		
		low	ns		
	**TLR3**	high	ns		
		low	ns		
	TLR4	high	ns		
		low	ns		
	TLR5	high	ns		
		low	ns		
	TLR6	high	ns		
		low	ns		
	**TLR7**	high	ns		
		low	ns		
	**TLR8**	high	ns		
		low	ns		
	**TLR9**	high	ns		
		low	ns		
	TLR10	high	ns		
		low	ns		
	**Gastric**				
	TLR1	high	0.00037	30.7	87
		low	ns		
	TLR2	high	0.00056	26.6	65
		low	ns		
	**TLR3**	high	0.012	32.6	53.7
		low	ns		
	TLR4	high	0.0015	15.2	24.23
		low	ns		
	TLR5	high	ns		
		low	0.011	26.2	46.8
	TLR6	high	ns		
		low	0.0064	36.4	89.43
	**TLR7**	high	0.00088	28	51.4
		low	ns		
	**TLR8**	high	0.00071	14.3	24.03
		low	ns		

**TLR9**	high	ns		
	low	0.0034	13	23.6
TLR10	high	ns		
	low	0.017	12.9	21.83
**Lung**				
TLR1	high	ns		
	low	1.70E-09	80	40.77
TLR2	high	ns		
	low	0.0019	61.3	46
**TLR3**	high	ns		
	low	0.00015	74	48
TLR4	high	ns		
	low	1.80E-06	102	52
TLR5	high	ns		
	low	0.02	66	51
TLR6	high	ns		
	low	1.20E-06	96.2	42
**TLR7**	high	ns		
	low	0.0053	63	46
**TLR8**	high	ns		
	low	3.10E-06	87.7	42
**TLR9**	high	ns		
	low	2.50E-05	100	52
TLR10	high	ns		
	low	1.50E-06	102	52
**Myeloma**				
TLR1	high	ns		
	low	2.50E-06	29.93	15.37
TLR2	high	3.30E-08	55	20.87
	low	ns		
**TLR3**	high	0.001	35	18.83
	low	0.00024	47	23.78
TLR4	high	0.0076	52.03	36.93
	low	ns		
TLR5	high	ns		
	low	2.60E-05	38.27	15
TLR6	high	ns		
	low	0.00082	55	32.14
**TLR7**	high	2.60E-06	47	19
	low	ns		
**TLR8**	high	5.30E-05	71	39.25

	low	ns		
**TLR9**	high	ns		
	low	0.00026	59	34
TLR10	high	ns		
	low	8.00E-04	49	25
**Ovarian**				
TLR1	high	ns		
	low	ns		
TLR2	high	ns		
	low	ns		
**TLR3**	high	0.038	53.33	45
	low	ns		
TLR4	high	ns		
	low	0.0086	50	36
TLR5	high	ns		
	low	ns		
TLR6	high	ns		
	low	ns		
**TLR7**	high	ns		
	low	ns		
**TLR8**	high	ns		
	low	0.00019	53.33	35
**TLR9**	high	ns		
	low	0.037	60.29	45
TLR10	high	ns		
	low	0.0035	69	40
**HCC Caucasian**				
TLR1	high	ns		
	low	0.018	71.03	27.57
TLR2	high	ns		
	low	0.0036	71.03	29.97
**TLR3**	high	ns		
	low	ns		
TLR4	high	ns		
	low	0.0077	56.47	27.57
TLR5	high	ns		
	low	0.036	59.7	31.03
TLR6	high	ns		
	low	0.016	59.7	31.03
**TLR7**	high	ns		
	low	0.021	59.7	27.57

	**TLR8**	high	ns		
		low	0.004	59.7	29.97
	**TLR9**	high	ns		
		low	ns		
	TLR10	high	ns		
		low	ns		
